# The genome sequence of the plain dark bee, *Stelis phaeoptera* (Kirby, 1802)

**DOI:** 10.12688/wellcomeopenres.18876.1

**Published:** 2023-01-27

**Authors:** Clare Boyes, University of Oxford and Wytham Woods Genome Acquisition Lab, Darwin Tree of Life Barcoding collective, Wellcome Sanger Institute Tree of Life programme, Wellcome Sanger Institute Scientific Operations: DNA Pipelines collective, Tree of Life Core Informatics collective, Darwin Tree of Life Consortium

**Affiliations:** 1 Independent Researcher, Wytham, UK

**Keywords:** Stelis phaeoptera, plain dark bee, genome sequence, chromosomal; Hymenoptera

## Abstract

We present a genome assembly from an individual female *Stelis phaeoptera* (the plain dark bee; Arthropoda; Insecta; Hymenoptera; Megachilidae). The genome sequence is 301 megabases in span. Most of the assembly is scaffolded into 17 chromosomal pseudomolecules. The mitochondrial genome has also been assembled and is 18.6 kilobases in length. Gene annotation of this assembly on Ensembl identified 9,850 protein coding genes.

## Species taxonomy

Eukaryota; Metazoa; Ecdysozoa; Arthropoda; Hexapoda; Insecta; Pterygota; Neoptera; Endopterygota; Hymenoptera; Apocrita; Aculeata; Apoidea; Megachilidae; Megachilinae; *Stelis*; *Stelis phaeoptera* (Kirby, 1802) (NCBI:txid2249760).

## Background

*Stelis phaeoptera* (plain dark bee) is a rare megachilid bee, found across the western Palaearctic from Spain and North Africa, to Kazakhstan. In Britain, the species is found in scattered locations across Wales and the south of England. The species, which was never common, has declined in the last 30 years, particularly in the south of England ( Else & Edwards, 2018). However, it has a stronghold in the Welsh and Shropshire Marches ( Jones & Cheeseborough, 2012).

As its common name suggests, the appearance of this small bee is unremarkable, having a dark, strongly punctate, body ( Falk & Lewington, 2015). It has one generation each year, with adults on the wing from late May to mid-August, and occasionally into September ( Else & Edwards, 2018). Bees in the genus *Stelis* are cleptoparasites and lay their eggs in the nests of other species of megachilid bee. They are most often seen around garden ‘bee hotels’, as these are favoured by their host species. In the UK, confirmed hosts are the mason bees, *Osmia leaiana* ( Jones & Cheeseborough, 2012), and *Osmia bicornis* ( Boyes, 2021; Boyes, 2022). In Britain, *O. leaiana* appears to be the usual host, as its flight period coincides with *S. phaeoptera*. The flight period of *O. bicornis* is earlier, only overlapping by about a week, leaving fewer opportunities for *S. phaeoptera* to use this species. Interestingly, *O. leiana* sometimes exhibits a two-year life cycle ( Boyes, 2020), which may be a strategy to reduce parasitism as the *Stelis* hatch after one year, leaving fewer hosts available.

The genome of *S. phaeoptera* was sequenced as part of the Darwin Tree of Life Project, a collaborative effort to sequence all named eukaryotic species in the Atlantic Archipelago of Britain and Ireland. Here we present a chromosomally complete genome sequence for *S. phaeoptera*, based on one female specimen from a garden in Powys, UK.

## Genome sequence report

**Figure 1.**
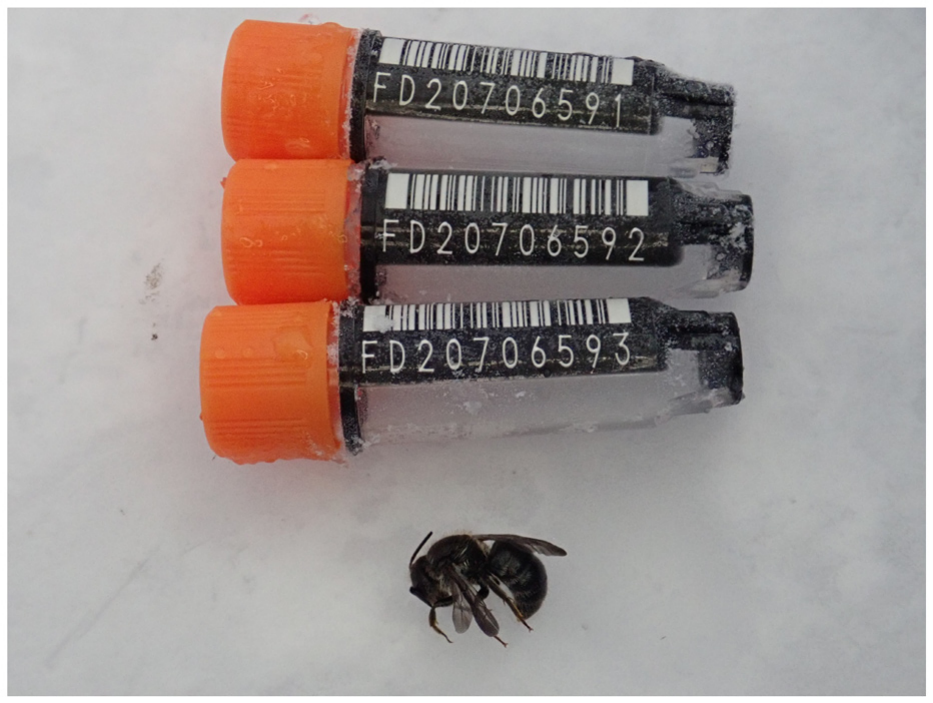
Photograph of the *Stelis phaeoptera* (iyStePhae1) specimen used for genome sequencing.

The genome was sequenced from one female *Stelis phaeoptera* ( Figure 1) collected from a garden in Middletown, Powys, UK (latitude 52.70, longitude –3.03). A total of 79-fold coverage in Pacific Biosciences single-molecule HiFi long reads was generated. Primary assembly contigs were scaffolded with chromosome conformation Hi-C data. Manual assembly curation corrected six missing joins or misjoins, reducing the scaffold number by 2,15% and increasing the scaffold N50 by 2.25%.

**Figure 2.**
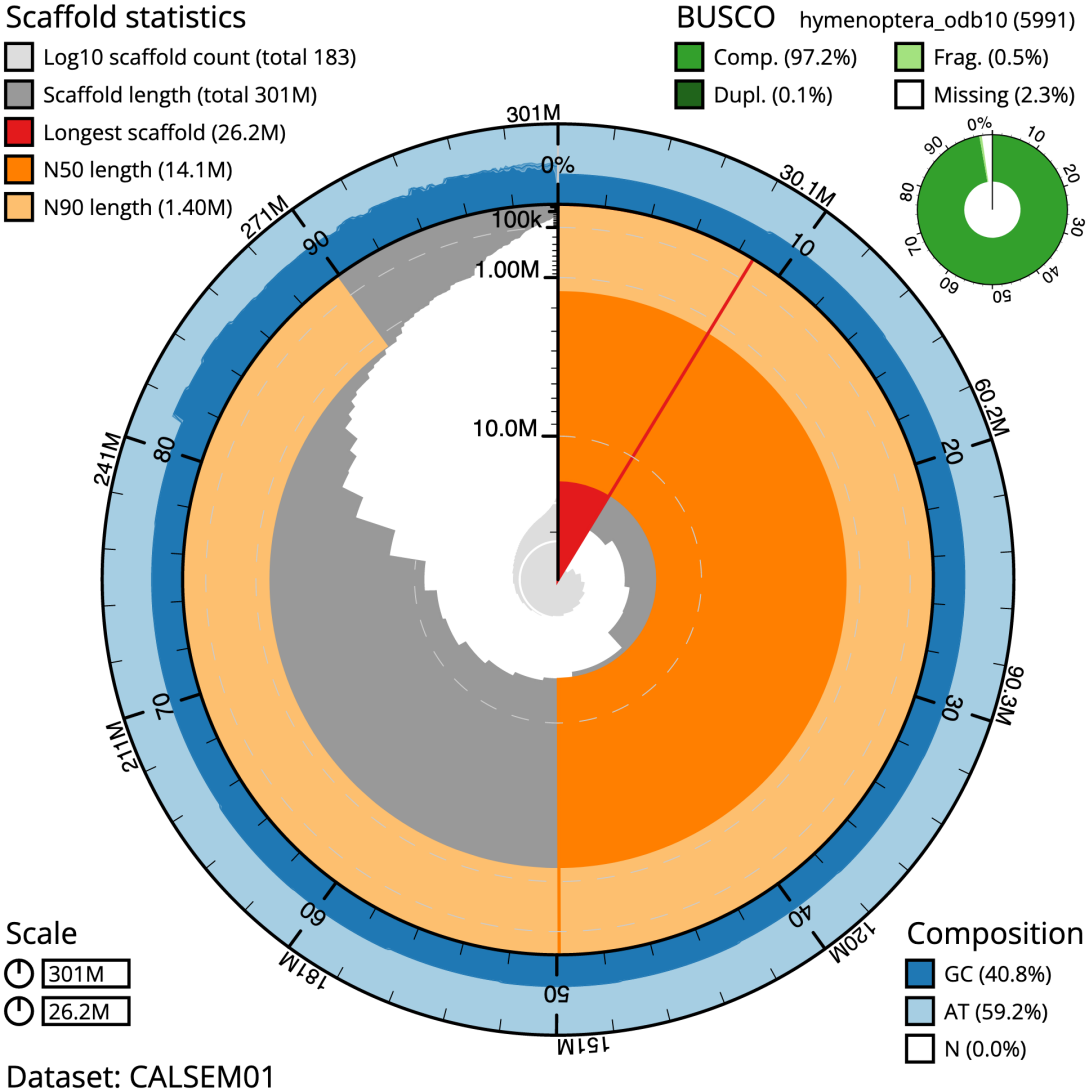
Genome assembly of *Stelis phaeoptera*, iyStePhae1.1: metrics. The BlobToolKit Snailplot shows N50 metrics and BUSCO gene completeness. The main plot is divided into 1,000 size-ordered bins around the circumference with each bin representing 0.1% of the 301,081,373 bp assembly. The distribution of scaffold lengths is shown in dark grey with the plot radius scaled to the longest scaffold present in the assembly (26,202,834 bp, shown in red). Orange and pale-orange arcs show the N50 and N90 sequence lengths (14,114,627 and 1,402,165 bp), respectively. The pale grey spiral shows the cumulative scaffold count on a log scale with white scale lines showing successive orders of magnitude. The blue and pale-blue area around the outside of the plot shows the distribution of GC, AT and N percentages in the same bins as the inner plot. A summary of complete, fragmented, duplicated and missing BUSCO genes in the hymenoptera_odb10 set is shown in the top right. An interactive version of this figure is available at https://blobtoolkit.genomehubs.org/view/iyStePhae1.1/dataset/CALSEM01/snail.

The final assembly has a total length of 301.0 Mb in 182 sequence scaffolds, with a scaffold N50 of 14.1 Mb ( Table 1). Most (81.34%) of the assembly sequence was assigned to 17 chromosomal-level scaffolds. Chromosome-scale scaffolds confirmed by the Hi-C data are named in order of size ( Figure 2– Figure 5; Table 2). A large number of heterochromatic contigs remain unlocalised ( Figure 5). The assembly has a BUSCO v5.3.2 ( Manni *et al.*, 2021) completeness of 97.2% (single 97.1%, duplicated 0.1%) using the OrthoDB v10 Hymenoptera reference set (n = 5,991). While not fully phased, the assembly deposited is of one haplotype. Contigs corresponding to the second haplotype have also been deposited.

**Table 1.** Genome data for *Stelis phaeoptera*, iyStePhae1.1.

Project accession data		
Assembly identifier	iyStePhae1.1	
Species	*Stelis phaeoptera*	
Specimen	iyStePhae1	
NCBI taxonomy ID	2249760	
BioProject	PRJEB52657	
BioSample ID	SAMEA10978746	
Isolate information	female: iyStePhae1 (PacBio: thorax; head: Hi-C	
Assembly metrics *		*Benchmark*
Consensus quality (QV)	64.4	*≥ 50*
*k*-mer completeness	100%	*≥ 95%*
BUSCO **	C:97.2%[S:97.1%,D:0.1%], F:0.5%,M:2.3%,n:5991	*C ≥ 95%*
Percentage of assembly mapped to chromosomes	81.34%	*≥ 95%*
Sex chromosomes	N/A	*localised homologous pairs*
Organelles	Mitochondrial genome assembled	*complete single alleles*
Raw data accessions		
PacificBiosciences SEQUEL II	ERR9793193	
Hi-C Illumina	ERR9714291	
PolyA RNA-Seq Illumina	ERR10123698	
Genome assembly		
Assembly accession	GCA_943735885.1	
*Accession of alternate haplotype*	GCA_943735895.1	
Span (Mb)	301.1	
Number of contigs	203	
Contig N50 length (Mb)	13.4	
Number of scaffolds	182	
Scaffold N50 length (Mb)	14.1	
Longest scaffold (Mb)	26.2	
Genome annotation		
Number of protein-coding genes	9,850	
Number of non-coding genes	1,969	
Number of gene transcripts	18,430	

* Assembly metric benchmarks are adapted from column VGP-2020 of “Table 1: Proposed standards and metrics for defining genome assembly quality” from ( Rhie *et al.*, 2021). ** BUSCO scores based on the hymenoptera_odb10 BUSCO set using v5.3.2. C = complete [S = single copy, D = duplicated], F = fragmented, M = missing, n = number of orthologues in comparison. A full set of BUSCO scores is available at https://blobtoolkit.genomehubs.org/view/iyStePhae1.1/dataset/CALSEM01/busco.

**Figure 3.**
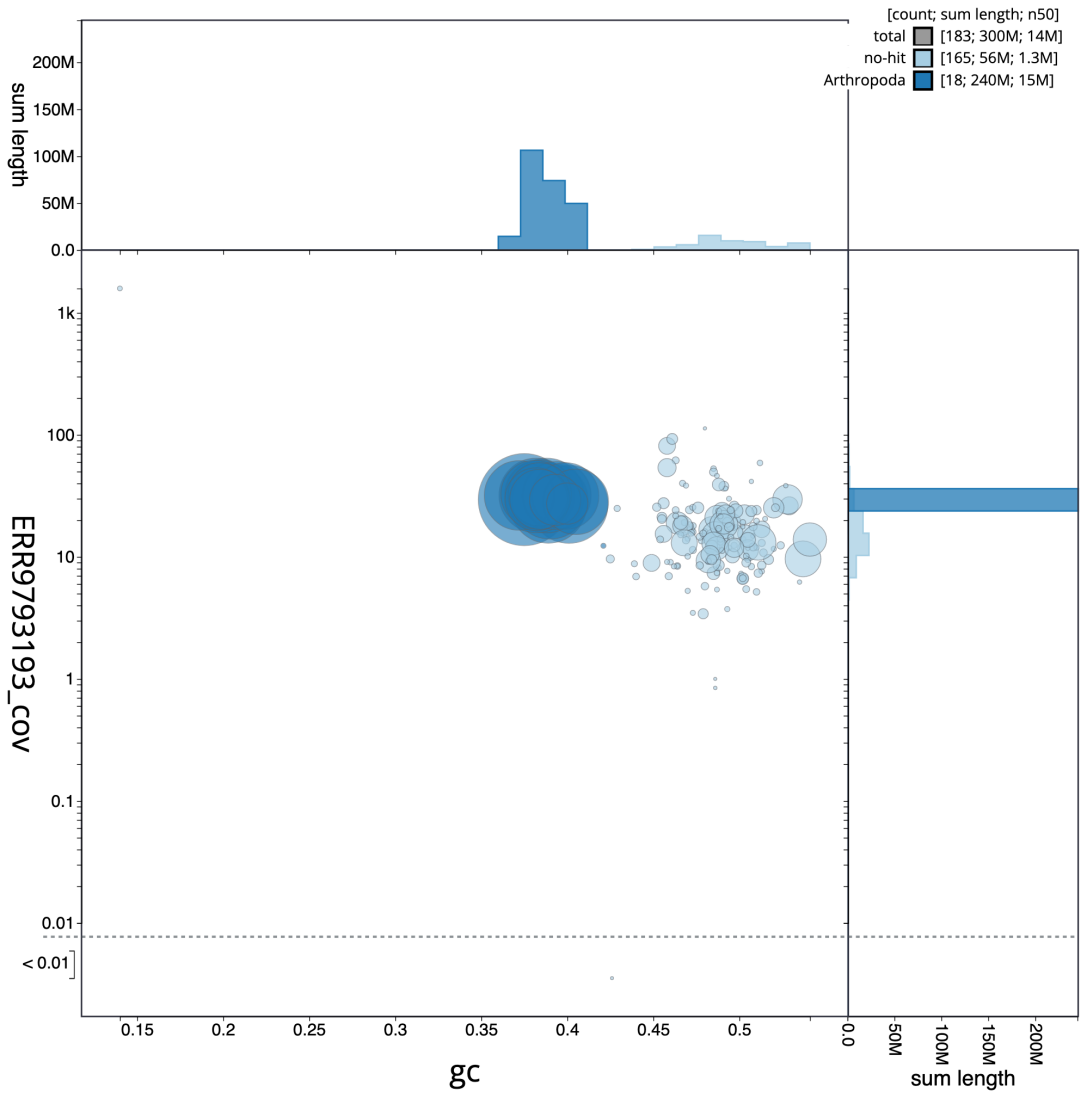
Genome assembly of *Stelis phaeoptera*, iyStePhae1.1: GC coverage. BlobToolKit GC-coverage plot. Scaffolds are coloured by phylum. Circles are sized in proportion to scaffold length. Histograms show the distribution of scaffold length sum along each axis. An interactive version of this figure is available at https://blobtoolkit.genomehubs.org/view/iyStePhae1.1/dataset/CALSEM01/blob.

**Figure 4.**
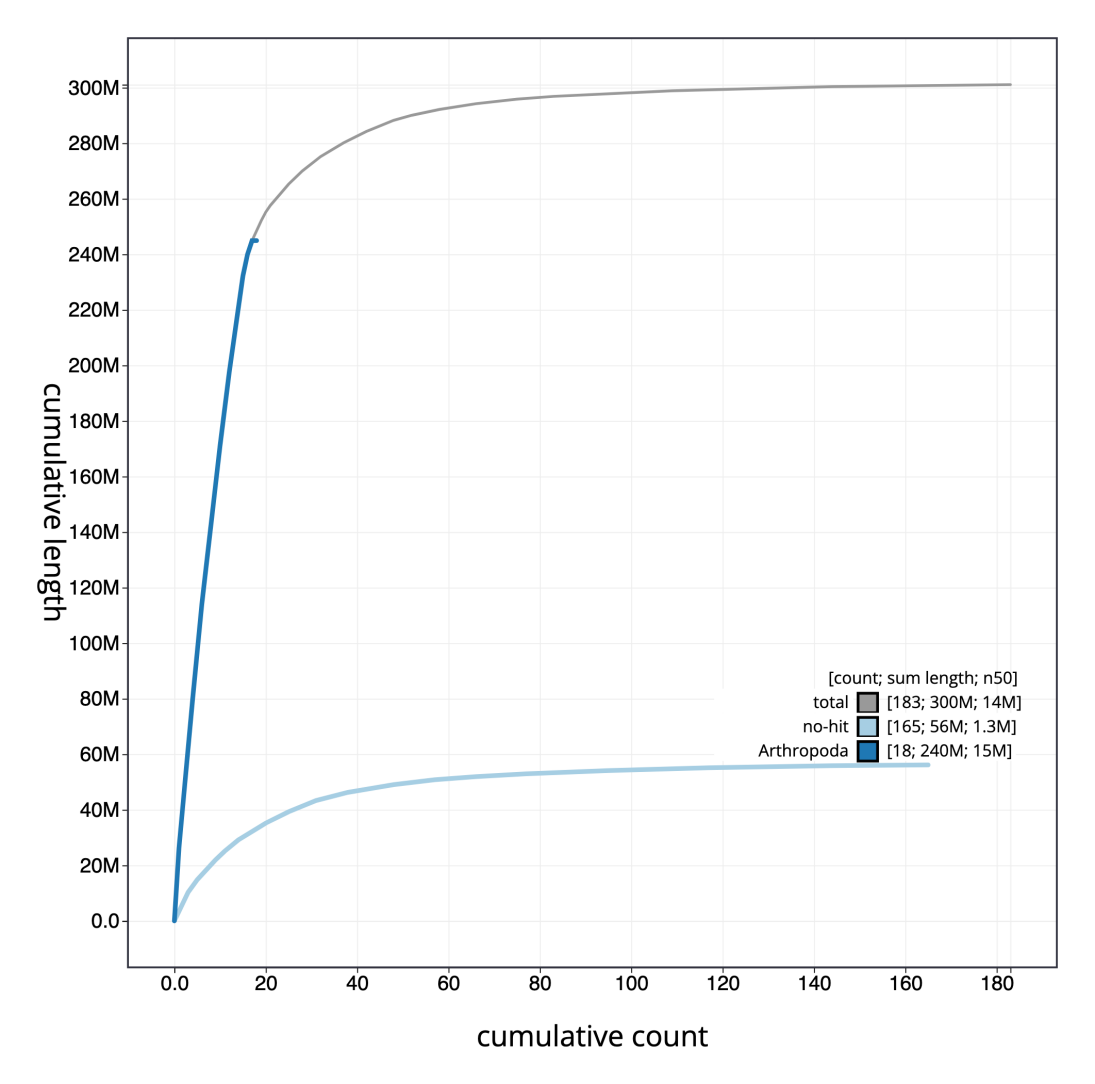
Genome assembly of *Stelis phaeoptera*, iyStePhae1.1: cumulative sequence. BlobToolKit cumulative scaffold plot. The grey line shows cumulative length for all scaffolds. Coloured lines show cumulative lengths of scaffolds assigned to each phylum using the buscogenes taxrule. An interactive version of this figure is available at https://blobtoolkit.genomehubs.org/view/iyStePhae1.1/dataset/CALSEM01/cumulative.

**Figure 5.**
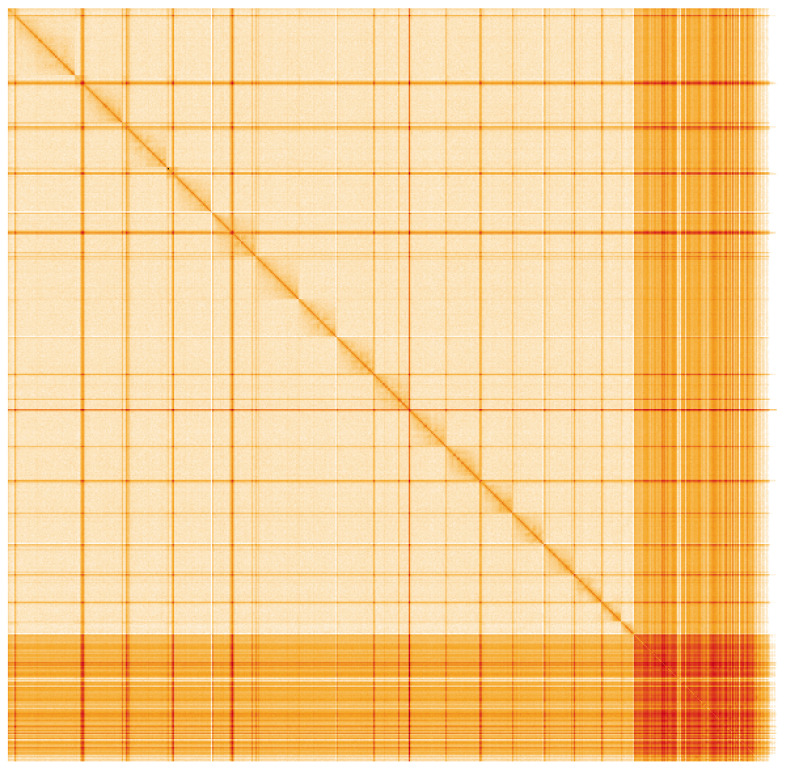
Genome assembly of *Stelis phaeoptera*, iyStePhae1.1: Hi-C contact map. Hi-C contact map of the iyStePhae1.1 assembly, visualised using HiGlass. Chromosomes are shown in order of size from left to right and top to bottom. An interactive version of this figure may be viewed at https://genome-note-higlass.tol.sanger.ac.uk/l/?d=a1cO2vziT4C_vIiOJ9DjQA.

**Table 2.** Chromosomal pseudomolecules in the genome assembly of *Stelis phaeoptera*, iyStePhae1.

INSDC accession	Chromosome	Size (Mb)	GC%
OX030930.1	1	26.2	37.5
OX030931.1	2	18.56	40.1
OX030932.1	3	17.61	38.9
OX030933.1	4	17.59	39
OX030934.1	5	16.99	38.8
OX030935.1	6	16.9	38.2
OX030936.1	7	14.68	37.2
OX030937.1	8	14.68	38.4
OX030938.1	9	14.11	39.4
OX030939.1	10	13.8	38.1
OX030940.1	11	13.4	39.9
OX030941.1	12	12.89	40.5
OX030942.1	13	12.13	38.4
OX030943.1	14	11.86	38.2
OX030944.1	15	10.78	38.4
OX030945.1	16	7.77	39.3
OX030946.1	17	4.94	40
OX030947.1	MT	0.02	14.1

## Genome annotation report

The *S. phaeoptera* genome assembly (GCA_943735885.1) was annotated using the Ensembl rapid annotation pipeline ( Table 1; https://rapid.ensembl.org/Stelis_phaeoptera_GCA_943735885.1/Info/Index). The resulting annotation includes 18,430 transcribed mRNAs from 9,850 protein-coding and 1,969 non-coding genes.

## Methods

### Sample acquisition and nucleic acid extraction

A female *Stelis phaeoptera* (iyStePhae1) was collected from a garden in Middletown, Powys, UK (latitude 52.70, longitude –3.03) by potting, on 4 July 2021. The specimen was collected and identified by Clare Boyes. The specimen was snap-frozen on dry ice.

DNA was extracted at the Tree of Life laboratory, Wellcome Sanger Institute (WSI). The iyStePhae1 sample was weighed and dissected on dry ice with head tissue set aside for Hi-C sequencing. Abdomen tissue was disrupted using a Nippi Powermasher fitted with a BioMasher pestle. High molecular weight (HMW) DNA was extracted using the Qiagen MagAttract HMW DNA extraction kit. Low molecular weight DNA was removed from a 20 ng aliquot of extracted DNA using 0.8X AMpure XP purification kit prior to 10X Chromium sequencing; a minimum of 50 ng DNA was submitted for 10X sequencing. HMW DNA was sheared into an average fragment size of 12–20 kb in a Megaruptor 3 system with speed setting 30. Sheared DNA was purified by solid-phase reversible immobilisation using AMPure PB beads with a 1.8X ratio of beads to sample to remove the shorter fragments and concentrate the DNA sample. The concentration of the sheared and purified DNA was assessed using a Nanodrop spectrophotometer and Qubit Fluorometer and Qubit dsDNA High Sensitivity Assay kit. Fragment size distribution was evaluated by running the sample on the FemtoPulse system.

RNA was extracted from thorax tissue of iyStePhae1 in the Tree of Life Laboratory at the WSI using TRIzol, according to the manufacturer’s instructions. RNA was then eluted in 50 μl RNAse-free water and its concentration was assessed using a Nanodrop spectrophotometer and Qubit Fluorometer using the Qubit RNA Broad-Range (BR) Assay kit. Analysis of the integrity of the RNA was done using Agilent RNA 6000 Pico Kit and Eukaryotic Total RNA assay.

### Sequencing

Pacific Biosciences HiFi circular consensus and 10X Genomics read cloud DNA sequencing libraries were constructed according to the manufacturers’ instructions. Poly(A) RNA-Seq libraries were constructed using the NEB Ultra II RNA Library Prep kit. DNA and RNA sequencing were performed by the Scientific Operations core at the WSI on Pacific Biosciences SEQUEL II (HiFi) and Illumina NovaSeq 6000 (RNA-Seq) instruments. Hi-C data were also generated from head tissue of iyStePhae1 using the Arima v2 kit and sequenced on the NovaSeq 6000 instrument.

### Genome assembly

Assembly was carried out with Hifiasm ( Cheng *et al.*, 2021) and haplotypic duplication was identified and removed with purge_dups ( Guan *et al.*, 2020). The assembly was scaffolded with Hi-C data ( Rao *et al.*, 2014) using YaHS ( Zhou *et al.*, 2022). The assembly was checked for contamination as described previously ( Howe *et al.*, 2021). Manual curation was performed using HiGlass ( Kerpedjiev *et al.*, 2018) and Pretext ( Harry, 2022). The mitochondrial genome was assembled using MitoHiFi ( Uliano-Silva *et al.*, 2022), which performed annotation using MitoFinder ( Allio *et al.*, 2020). The genome was analysed and BUSCO scores were generated within the BlobToolKit environment ( Challis *et al.*, 2020). Table 3 contains a list of all software tool versions used, where appropriate.

**Table 3.** Software tools and versions used.

Software tool	Version	Source
BlobToolKit	3.4.0	Challis *et al.*, 2020
Hifiasm	0.16.1-r375	Cheng *et al.*, 2021
HiGlass	1.11.6	Kerpedjiev *et al.*, 2018
MitoHiFi	2	Uliano-Silva *et al.*, 2022
PretextView	0.2	Harry, 2022
purge_dups	1.2.3	Guan *et al.*, 2020
YaHS	yahs-1.1.91eebc2	Zhou *et al.*, 2022

### Genome annotation

The Ensembl gene annotation system ( Aken *et al.*, 2016) was used to generate annotation for the *Stelis phaeoptera* assembly (GCA_943735885.1). Annotation was created primarily through alignment of transcriptomic data to the genome, with gap filling via protein to-genome alignments of a select set of proteins from UniProt ( UniProt Consortium, 2019).

### Ethics/compliance issues

The materials that have contributed to this genome note have been supplied by a Darwin Tree of Life Partner. The submission of materials by a Darwin Tree of Life Partner is subject to the Darwin Tree of Life Project Sampling Code of Practice. By agreeing with and signing up to the Sampling Code of Practice, the Darwin Tree of Life Partner agrees they will meet the legal and ethical requirements and standards set out within this document in respect of all samples acquired for, and supplied to, the Darwin Tree of Life Project. Each transfer of samples is further undertaken according to a Research Collaboration Agreement or Material Transfer Agreement entered into by the Darwin Tree of Life Partner, Genome Research Limited (operating as the Wellcome Sanger Institute), and in some circumstances other Darwin Tree of Life collaborators.

## Data Availability

European Nucleotide Archive: *Stelis phaeoptera* (plain dark bee). Accession number PRJEB52657; https://identifiers.org/ena.embl/PRJEB52657. ( Wellcome Sanger Institute, 2022) The genome sequence is released openly for reuse. The *Stelis phaeoptera* genome sequencing initiative is part of the Darwin Tree of Life (DToL) project. All raw sequence data and the assembly have been deposited in INSDC databases. Raw data and assembly accession identifiers are reported in Table 1.

## Author information

Members of the University of Oxford and Wytham Woods Genome Acquisition Lab are listed here: https://doi.org/10.5281/zenodo.4789928.

Members of the Darwin Tree of Life Barcoding collective are listed here: https://doi.org/10.5281/zenodo.4893703.

Members of the Wellcome Sanger Institute Tree of Life programme are listed here: https://doi.org/10.5281/zenodo.4783585.

Members of Wellcome Sanger Institute Scientific Operations: DNA Pipelines collective are listed here: https://doi.org/10.5281/zenodo.4790455.

Members of the Tree of Life Core Informatics collective are listed here: https://doi.org/10.5281/zenodo.5013541.

Members of the Darwin Tree of Life Consortium are listed here: https://doi.org/10.5281/zenodo.4783558.
