# Supplementary material for: The genome sequence of the plain dark bee, Stelis phaeoptera (Kirby, 1802)
Source: Wellcome Open Res. 2023 Jan 27;8:42. [Version 1] doi: 10.12688/wellcomeopenres.18876.1 (PMC10280034; doi:10.12688/wellcomeopenres.18876.1)
Supplement: Peer review history

# Peer review: Reviewer response for version 1

**DOI:** 10.21956/wellcomeopenres.20930.r58495

**Published:** 2023-06-12

**Authors:** Yannick Wurm

**Affiliations:** 1 The Alan Turing Institute, London, England, UK; 2 School of Biological and Behavioural Sciences, Queen Mary University of London, London, England, UK

The authors report the sequencing of a new bee genome. All looks fine and in line with high standards.

 Only few minor comments:

- "Manual assembly curation corrected six missing joins or misjoins, reducing the scaffold number" - how was this done? No explanation of manual criteria or references are provided.
- "2,15%" - unusual to have commas.
- "named in order of size". Decreasing?
- Table 1 "Raw data"- indicates NCBI project numbers. It would be great to see how much sequencing effort there is for each in this table.
- Fig 2: the unplaced scaffolds seem to have 10% higher GC. Is this usual? Are they potentially spread throughout the genome? Or represent one or few other chromosomes? Or contamination? Some interpretation would be welcome.

Are sufficient details of methods and materials provided to allow replication by others?

Partly

Is the rationale for creating the dataset(s) clearly described?

Yes

Are the datasets clearly presented in a useable and accessible format?

Yes

Are the protocols appropriate and is the work technically sound?

Yes

Reviewer Expertise:

Ant genome evolution, pollinator health, comparative genomics, bioinformatics.

I confirm that I have read this submission and believe that I have an appropriate level of expertise to confirm that it is of an acceptable scientific standard.

# Peer review: Reviewer response for version 1

**DOI:** 10.21956/wellcomeopenres.20930.r58501

**Published:** 2023-06-02

**Authors:** Natalia Araujo

**Affiliations:** 1 Unit of Evolutionary Biology & Ecology, Universite Libre de Bruxelles, Brussels, Brussels, Belgium

The authors report the assemblies of the nuclear and mitochondrial genomes of the bee *Stelis phaeoptera*. This megachilidae bee is a rare cleptoparasite of western Palearctic occurrence. Using a single female, they generated genomic sequencing data using long-read Pacbio Hi-Fi from the abdomen tissue, RNASeq transcriptomic data from the thorax, and Hi-C conformation data from the bee head. The nuclear genome length was 301Mb within 182 scaffolds and 17 chromosomal pseudomolecules encompassing 9,850 protein genes, while the mt genome size was 18.8kb.

 Overall, I have no major concerns about this manuscript report, the pipeline for data generation and analyses is well described and established by the Darwin Tree of Life consortium and it is a reference to the field. Genome annotation could benefit from using more tissues/ developmental stages to better represent species gene set. Still, I understand this is a common limitation and I recognize the value of protocol optimization (using only a single individual) employed in the study. I appreciate that diploid information was also deposited along with the reference genome. The assembly results fall within the expected values for bees, the figures are adequate, and the data is openly available. Also, considering this bee species is of rare occurrence and it has declined in at least part of its natural habitat the data is valuable for the community.

 I have only a few minor comments/ suggestions to this data note as follows:

1. Figure 1 could benefit from a scale. Although there are tubes in the figure that can be used as a reference it is hard to tell the size of the bee only from that.
2. Authors say that 79-fold coverage of data was generated from the HiFi sequencing, but there is no information about the coverage, or the amount of data, generated for the Hi-C and RNASeq. How many reads were produced in these sequencings?
3. I know the data note is focused on the nuclear genome assembly, but since the mitochondrial genome assembly is also reported more information about this assembly could be included. For example, the size of the mtgenome is only reported in the abstract, but not in the results section. There is also no information about the resulting annotation of the mtgenome. I don’t believe much detail is needed, but were all genes present in the mtgenome assembled as expected? Was the AT rich region complete and the circular molecule recovered? Maybe a figure representing/ summarizing this genome could also be useful?

Are sufficient details of methods and materials provided to allow replication by others?

Partly

Is the rationale for creating the dataset(s) clearly described?

Yes

Are the datasets clearly presented in a useable and accessible format?

Yes

Are the protocols appropriate and is the work technically sound?

Yes

Reviewer Expertise:

Genomics, Transcriptomics, Evolution

I confirm that I have read this submission and believe that I have an appropriate level of expertise to confirm that it is of an acceptable scientific standard.
